# Correcting systematic bias and instrument measurement drift with mzRefinery

**DOI:** 10.1093/bioinformatics/btv437

**Published:** 2015-08-04

**Authors:** Bryson C. Gibbons, Matthew C. Chambers, Matthew E. Monroe, David L. Tabb, Samuel H. Payne

**Affiliations:** 1 ^1^Biological Sciences Division, Pacific Northwest National Laboratory, Richland WA 99354 and; 2 ^2^Department of Biomedical Informatics, Vanderbilt University School of Medicine, Nashville, TN 37232, USA

## Abstract

**Motivation:** Systematic bias in mass measurement adversely affects data quality and negates the advantages of high precision instruments.

**Results**: We introduce the mzRefinery tool for calibration of mass spectrometry data files. Using confident peptide spectrum matches, three different calibration methods are explored and the optimal transform function is chosen. After calibration, systematic bias is removed and the mass measurement errors are centered at 0 ppm. Because it is part of the ProteoWizard package, mzRefinery can read and write a wide variety of file formats.

**Availability and implementation:** The mzRefinery tool is part of msConvert, available with the ProteoWizard open source package at http://proteowizard.sourceforge.net/

**Contact:** samuel.payne@pnnl.gov

**Supplementary information:** Supplementary data are available at *Bioinformatics* online.

## 1 Introduction

For data analysis algorithms to take advantage of the higher accuracy of newer mass spectrometers, it is essential to remove systematic bias in mass measurement. Mass measurement error may originate from a variety of sources, e.g. power supply voltage/temperature drift, space charge effects, temperature/humidity variation in the laboratory, vacuum system stability, etc. Real-time calibration adjusts the mass measurement during data acquisition (Charles, 2003; Olsen *et al.,* 2005;), typically using a known species as an internal reference. Lock mass methods may also be used to calibrate after the run has completed (Zhang *et al.,* 2011). A separate method for calibration utilizes spectrum identifications to estimate measurement error and guide mass correction (Cox *et al.,* 2011; Petyuk *et al.,* 2010).

We present a new calibration tool, mzRefinery, written directly into the ProteoWizard package (Kessner *et al.,* 2008). Like existing tools, mzRefinery models mass measurement error based on peptide identifications and finds the optimal calibration function. In addition to simply adjusting the precursor ion, mzRefinery corrects the *m*/*z* of every ion in any high-resolution spectrum. With the increasingly common use of high-resolution tandem mass spectra in PRM and DIA experiments, more data are being created with high-resolution fragments. Given the inherent complexity of such multiplexed fragmentation protocols, calibrating the mass accuracy will be a great benefit for these experiments.

## 2 Implementation

mzRefinery has three different methods for calibration. The goal of each method is to identify the *m*/*z* offset that should be applied in creating a calibrated spectrum file. The software architecture is specifically designed to allow for new calibration methods to be written and seamlessly integrated. A detailed description of the mass spectrometry data files, software class architecture and operation are provided in Supplementary Data and Supplementary Figure S1.

### 2.1 Global shift

Using the sub-class AdjustSimpleGlobal creates a single global shift. For every confident identification in the mzIdentML file (default *q* < 0.01), the exact monoisotopic *m*/*z* is calculated and compared with the observed *m*/*z* (using xml field experimentalMassToCharge). Mass errors >±0.2 *m*/*z* are filtered to avoid using data where the monoisotope was incorrectly reported by the spectrum file. After converting the error to ppm, the errors are collected into 0.5 ppm bins. After the entire file is processed, the median ppm error is calculated and used as the global shift. In the output mzML file, the SpectrumList_mzRefiner object applies the global ppm error to every peak in every high-resolution spectrum.

### 2.2 LC-dependent shift

Calculating the LC-dependent shift uses sub-class AdjustByScanTime. In general, the process is very similar to the calculation of a global shift. For every confident identification, both the ppm error and LC time are calculated. LC time is derived from the ScanStartTime field in mzIdentML, or from ScanStartTime in mzML. Errors are ordered by time and sorted into bins containing all scans within a 75-s period. The median ppm error of the bin is calculated, and smoothed using the median of neighboring bins (Supplementary Fig. S2). Bins in addition to the *i* + 1 and *i* − 1 neighbors are included as necessary to achieve a minimum of 100 identifications in the weighted average. When writing out the calibrated mzML file, the applied mass correction is generated through a linear interpolation of the median error values based on the scan time. By binning the data and then preforming a linear fit, the algorithm approximates a more complex smoothing.

### 2.3 *m*/*z*-dependent shift

Calculating the *m*/*z*-dependent shift uses sub-class AdjustByMass. This function is exactly like the LC-dependent shift except that measured *m*/*z* is tracked as the dependent variable.

## 3 Results and discussion

**Fig. 1.**
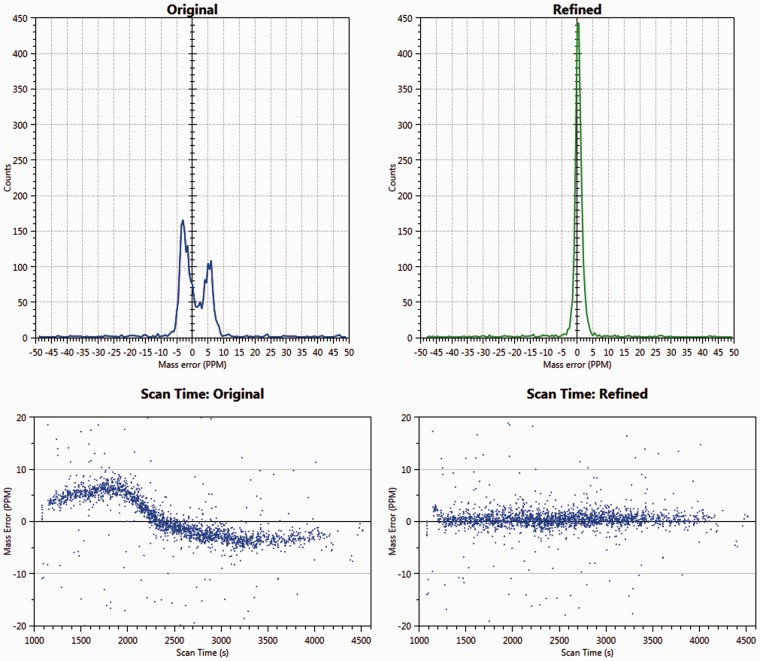
Calibration. The top two graphs show a histogram of mass error, calculated using PSM identifications for dataset sample3-B_BB4_01_926. This particular file has a bimodal error in the original. After calibration (top right), the error has been removed. The bottom two graphs plot mass measurement error according to scan number. The original data (bottom left) show that the error varies dramatically with time. By using the LC-dependent calibration, the errors are removed (bottom right)

The mzRefinery program is designed to calibrate any mass spectrometry data file based on a preliminary set of identifications. The algorithm is implemented within the msconvert program, part of the ProteoWizard suite, and therefore natively understands multiple input and output formats (Chambers *et al.,* 2012). As described in the Supplementary Data, we use mzRefinery to calibrate MS and MS/MS data from Thermo Orbitrap and Bruker QqTOF instruments. All files were searched with the appropriate database and parameters by both MSGF+ and MyriMatch. This preliminary set of PSMs was used as input to the msconvert program. The resulting mzML file has updated (calibrated) *m*/*z* values. Figure 1 shows the mass measurement error present in the original mzML files. We note that for the file in Figure 1, the error changed during the LC run, and is effectively eliminated by mzRefinery. The calibrated file shows no such dependency.

When viewing the performance of the algorithm across multiple files, it is remarkably consistent. For the 91 files tested, the original median error of any given file ranged between −2.8 and +8.4 ppm (average 1.4, SD 2.3). After calibration the median error ranged between −0.59 and +0.28 (average 0.02, SD 0.08), with 70 of the 91 files having a median error <±0.05 ppm. Thus, the method accurately removes any systematic bias in mass measurement.

A primary goal of the project is to make the mzRefinery algorithm broadly accessible. As part of the ProteoWizard suite, it is available as both an executable program and a platform for further development. The software architecture is intentionally written to be extensible and new calibration methods are automatically considered. Several reasons might prompt design of a new calibration method. In the current implementation, only one dependent variable is considered (i.e. LC time or *m*/*z*). However, previous study has shown that additional improvement is possible with more complex multivariate dependencies (Petyuk *et al.,* 2010). A second motivation would be to create a new calibration for a distinct instrument or mass analyzer. Although the current software has been shown to perform well on both Orbitrap and TOF instruments, we acknowledge that keeping up with new instrumentation is an ongoing process.

A suggested workflow for using mzRefinery is to first search each LC-MS/MS dataset for PSMs using fully tryptic search rules, no dynamic modifications and a relatively wide parent ion mass window, e.g. ±50 ppm. These parameters allow the search engine to quickly search for confident PSMs, yet allow for identifying PSMs even if the data were acquired when the instrument was not at its optimal calibration. Next, use mzRefinery to recalibrate each dataset using the identified PSMs from this initial search. Now re-search for PSMs in the data, but this time using the calibrated mzML files, a partially tryptic search, dynamic modifications and a narrower parent ion mass window, e.g. ±10 ppm or even ±ppm. Use of this narrow mass window will result in fewer false positives at a given false discovery rate.

## Supplementary Material

Supplementary Data
