# Supplementary material for: Correcting systematic bias and instrument measurement drift with mzRefinery
Source: Bioinformatics. 2015 Aug 4;31(23):3838–40. doi: 10.1093/bioinformatics/btv437 (PMC4653383; doi:10.1093/bioinformatics/btv437)
Supplement: Supplementary Data [file supp_31_23_3838__index.html]

Correcting systematic bias and instrument measurement drift with mzRefinery — Correcting systematic bias and instrument measurement drift with mzRefinery — Correcting systematic bias and instrument measurement drift with mzRefinery — Supplementary Data 

# Correcting systematic bias and instrument measurement drift with mzRefinery

## Supplementary Data

files

- Supplementary Data - docx file
