# Supplementary material for: Correcting systematic bias and instrument measurement drift with mzRefinery
Source: Bioinformatics. 2015 Aug 4;31(23):3838–40. doi: 10.1093/bioinformatics/btv437 (PMC4653383; doi:10.1093/bioinformatics/btv437)
Supplement: Supplementary Data [file supp_btv437_mzRefinerySupplement.docx]

| Application Note  Correcting systematic bias and instrument measurement drift with mzRefinery  Bryson C Gibbons^1^, Matthew C Chambers^2^, Matthew E Monroe^1^, David L Tabb^2^  and Samuel H Payne^1,*^  ^1^Biological Sciences Division, Pacific Northwest National Laboratory, Richland WA 99354 USA  ^2^Department of Biomedical Informatics, Vanderbilt University School of Medicine, Nashville, TN 37232, USA  Received on XXXXX; revised on XXXXX; accepted on XXXXX  Associate Editor: XXXXXXX |
| --- |

Supplemental Data

Class Architecture. As shown in Supplemental Figure 1, mzRefinery consists of several classes only one of which is exposed to the framework. The base class for calibration is AdjustmentObject. This abstract class serves to generalize the interface to specific calibration methods and requires that any inheriting classes implement two methods, shift and calculate.

mzRefinery is utilized by msconvert when creating a spectrum file, triggered by the command-line option for a filter type ‘mzRefiner’. Users must include a PSM identification file in a format supported by ProteoWizard (e.g. mzIdentML), to serve as the basis for mass calibration. The software automatically determines which of the three calibration methods is the best and applies it to the output mzML file. Although, we often refer to mzML and mzIdentML files, we note that ProteoWizard is able to use/create a wide variety of formats.

Datasets and Analyses. In this manuscript we use three groups of LC-MS/MS files: 90 Thermo LTQ Orbitrap files from whole-cell lysates of the cyanobacterium Cyanothece 8801; 12 Thermo QExactive files from the iPRG 2015 study of whole cell S. cerevisiae; and 12 Bruker QqTOF files from the iPRG 2015 study. All files were converted from the vendor specific format to mzML using msconvert as follows:

~> msconvert.exe "nativeFilePath" -o "OutputDirectory" --32 --filter "peakPicking true 1-"

mzML files were searched with MSGF+ using appropriate protein databases. MSGF+ parameters were:

~> java -Xmx4000M -jar MSGFPlus.jar -s "mzMLFilePath.mzML" -d "FastaFilePath.fasta" -t 20ppm -ti "-1,2" -ntt 1 -tda 1 -mod "Mods.txt" -minLength 7 -maxCharge 5

In this execution of MSGF+, Mods.txt contained a maximum of 0 mods for all datasets, with the exception of the iPRG 2015 Bruker QqTOF dataset, with was set to a maximum of 2 mods, with static Carbamidomethyl C, dynamic Oxidation M, and dynamic Protein N-term Acetylation.

mzML files were also searched with MyriMatch, to ensure that the mzRefinery functionality was generalizable. MyriMatch used these parameters:

~> myrimatch.exe -OutputFormat "mzIdentML" -workdir iPRG_2015\ -StaticMods "C 57.021463735" -DynamicMods "M * 15.99491463 ( ^ 42.01056467" -PrecursorMzToleranceRule "mono" -ProteinDatabase iPRG2015.fasta iPRG_2015\*.mzML

The mzid output was used to re-calibrate the mzML file using msconvert as follows:

~> msconvert.exe “mzMLFilePath.mzML” -o "OutputDirectory" -e "_FIXED.mzML" --32 --filter "mzRefiner ‘mzidFilePath.mzid’ msLevels=1- thresholdScore=MS-GF:SpecEValue thresholdValue=-1e-10"

All data is available on the MassIVE proteomics repository using the identifier MSV000079067, and proteomeXchange accession PXD001886.


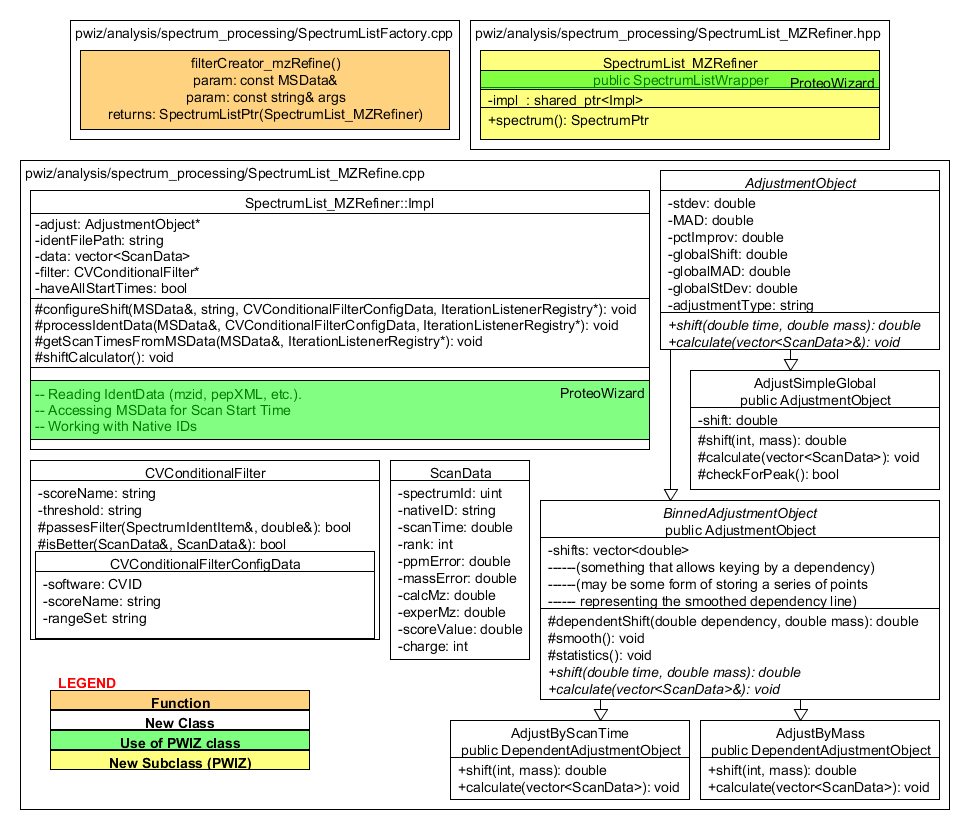


**Supplemental Figure 1 – Software architecture.** The class diagram of the mzRefinery code and how it fits into the proteowizard package. The main addition is a new .cpp/hpp file pair for SpectrumList_MZRefiner, which is called from the SpectrumListFactory object. The adjust variable within this class is an abstract object, AdjustmentObject. This abstract class is implemented in three classes each coding for a distinct method of calibration: AdjustSimpleGlobal, AdjustByMass and AdjustByScanTime.


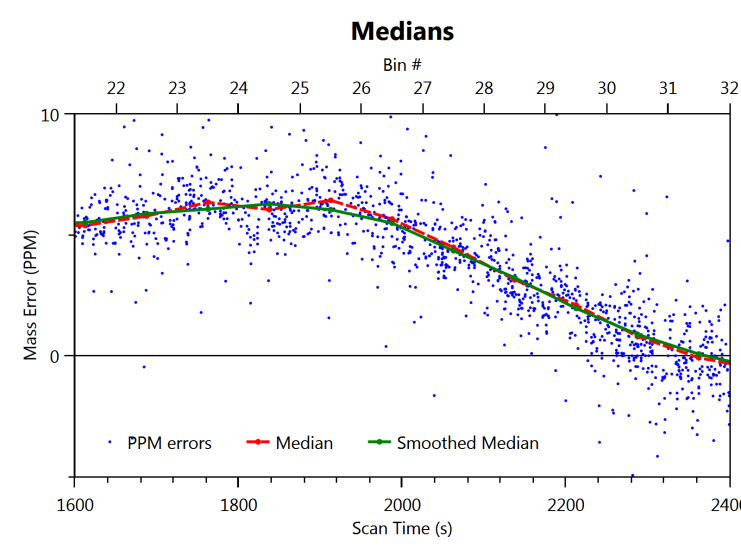


Supplemental Figure 2 - Identifying correction offset. The ppm error of each individual PSM is calculated (blue dots) and plotted according to time. Groups of PSMs are binned (see main text) and the median ppm error for a bin is calculated (red). The median error is adjusted by smoothing the data point with neighboring bins (green).
